# *Artemisia annua* L. plants lacking *Bornyl diPhosphate Synthase* reallocate carbon from monoterpenes to sesquiterpenes except artemisinin

**DOI:** 10.3389/fpls.2022.1000819

**Published:** 2022-10-12

**Authors:** Tomasz Czechowski, Caroline Branigan, Anne Rae, Deborah Rathbone, Tony R. Larson, David Harvey, Theresa M. Catania, Dong Zhang, Yi Li, Melissa Salmon, Dianna J. Bowles, Paul O´Maille, Ian A. Graham

**Affiliations:** ^1^ Centre for Novel Agricultural Products, Department of Biology, University of York, Heslington, York, United Kingdom; ^2^ Cherry Valley Farms Ltd, Cherry Valley House, Unit 1 Blossom Avenue, Humberston, North East Lincolnshire, United Kingdom; ^3^ Biorenewables Development Centre, 1 Hassacarr Close, Chessingham Park, Dunnington, York, United Kingdom; ^4^ College of Agriculture, South China Agricultural University, Guangzhou, China; ^5^ Department of Metabolic Biology, John Innes Centre, Norwich Research Park, Norwich, United Kingdom; ^6^ Patron Lab, Earlham Institute, Norwich Research Park, Norwich, Norfolk, United Kingdom; ^7^ SRI International, 333 Ravenswood Avenue, Menlo Park, CA, United States

**Keywords:** artemisia annua, artemisinin biosynthesis, camphor biosynthesis, bornyl diphosphate synthase, glandular secretory trichomes

## Abstract

The monoterpene camphor is produced in glandular secretory trichomes of the medicinal plant *Artemisia annua*, which also produces the antimalarial drug artemisinin. We have found that, depending on growth conditions, camphor can accumulate at levels ranging from 1- 10% leaf dry weight (LDW) in the Artemis F1 hybrid, which has been developed for commercial production of artemisinin at up to 1% LDW. We discovered that a camphor null (camphor-0) phenotype segregates in the progeny of self-pollinated Artemis material. Camphor-0 plants also show reduced levels of other less abundant monoterpenes and increased levels of the sesquiterpene precursor farnesyl pyrophosphate plus sesquiterpenes, including enzymatically derived artemisinin pathway intermediates but not artemisinin. One possible explanation for this is that high camphor concentrations in the glandular secretory trichomes play an important role in generating the hydrophobic conditions required for the non-enzymatic conversion of dihydroartemisinic acid tertiary hydroperoxide to artemisinin. We established that the camphor-0 phenotype associates with a genomic deletion that results in loss of a *Bornyl diPhosphate Synthase* (*AaBPS*) gene candidate. Functional characterization of the corresponding enzyme *in vitro* confirmed it can catalyze the first committed step in not only camphor biosynthesis but also in a number of other monoterpenes, accounting for over 60% of total volatiles in *A. annua* leaves. This *in vitro* analysis is consistent with loss of monoterpenes in camphor-0 plants. The *AaBPS* promoter drives high reporter gene expression in *A. annua* glandular secretory trichomes of juvenile leaves with expression shifting to non-glandular trichomes in mature leaves, which is consistent with *AaBPS* transcript abundance.

## Introduction

Malaria still poses a global threat, with 229 million cases occurring worldwide and 409,000 deaths in 2018 World Malaria Report (2019). Artemisinin, the main component in the WHO recommended treatment for malaria, is produced in glandular secretory trichomes (GSTs), specialized 10-cell structures found on the surface of the leaves, stems and flower buds of *Artemisia annua* L. More recent work suggests that the non-glandular trichome cells also express artemisinin biosynthetic pathway genes and produce artemisinin (Judd et al., 2019). The demand for the plant-sourced drug has been responded to by breeding efforts to improve yields, including the development of F1 hybrids such as Artemis (Delabays et al., 2001). Recently, over eighty additional natural products have been NMR-characterized from *A. annua*, including monoterpenes, sesquiterpenes, diterpenes, triterpenes/sterols, phenylpropanoids, flavonoids, aliphatic hydrocarbons, aromatic and aliphatic alcohols, aldehydes, ketones and acids (Czechowski et al., 2018). *A. annua* essential oil is synthesized in GSTs and has been the subject of numerous studies reporting antibacterial and antifungal activities, but chemical composition varies widely depending on the phytogeographic origin of the plants. Generally, the five major constituents in essential oil across *A. annua* varieties are artemisia ketone (2-68%), camphor (3-48%), 1,8-cineole (5-31.5%), germacrene D (0.3-21.2%) and borneol (7-20%) (Bilia et al., 2014). Camphor, traditionally obtained through the distillation of the wood of the camphor tree (*Cinnamomum camphora*), is a major essential oil component of many aromatic plant species. In addition to its use as a skin penetration enhancer (Chen et al., 2013), camphor also exhibits insecticidal, antimicrobial, antiviral, anticoccidial, anti-nociceptive, anticancer and antitussive activities (Chen et al., 2013). The camphor biosynthetic pathway begins with the cyclisation of geranyl diphosphate (GPP) by the enzyme (+)-bornyl diphosphate synthase (BPS), yielding (+)-bornyl diphosphate, which is then hydrolyzed to (+)-borneol through the action of bornyl-diphosphate diphosphatase. The last step is catalyzed by (+)-borneol dehydrogenase (BDH) as it oxidizes (+)-borneol to (+)-camphor (Croteau and Karp, 1976; Croteau and Karp, 1979a; Croteau and Karp, 1979b; Croteau et al., 1981). (+)-bornyl diphosphate synthases have been functionally characterized from sage, lavender and *Lippia dulcis* (Wise et al., 1998; Despinasse et al., 2017; Hurd et al., 2017; Singh et al., 2020). Sage Bornyl diPhosphate Synthase (SoBPS) has been cloned and structurally characterized revealing the exact molecular mechanism of GPP cyclisation to bornyl –diphosphate (Wise et al., 1998; Whittington et al., 2002). Interestingly, recombinant SoBPS is also able to produce significant amounts of other monoterpenes including camphene, limonene, a-pinene, terpinolene and myrcene (Wise et al., 1998). ^13^C isotope labelling studies have shown the GPP used for biosynthesis of camphor is produced through the non-mevalonate (MEP) pathway by combination of the C5 isoprenoid units, isopentenyl pyrophosphate (IPP) and dimethylallyl pyrophosphate (DMAPP) (Croteau et al., 1981). The pool of isoprenoid precursors is also required for the production of artemisinin, where IPP and DMAPP are of mixed biosynthetic origin, coming from both the cytosolic mevalonate and plastidial MEP pathways (Schramek et al., 2010). Camphor biosynthesis may therefore represent one of the major sinks for the plastidial pool of IPP and DMAPP in *A. annua* GSTs and if this is the case then a block in camphor synthesis could lead to increased production of artemisinin.

Detailed metabolite profiling of leaf material from an *A. annua* cv. Artemis F1 hybrid identified camphor as the most abundant volatile compound. Screening genetic variation led to the discovery of a camphor-0 phenotype in both Artemis F2 material and Artemis M2 material, that had been derived from selfed ethyl methanesulfonate (EMS) mutagenised Artemis M1 material. This allowed us to assess the impact of the removal of camphor, and the majority of monoterpenes, on the accumulation of artemisinin. Contrary to our expectations and despite a significant increase in farnesyl pyrophosphate (FPP, the precursor of the artemisinin biosynthetic pathway), and a number of pathway intermediates, artemisinin levels were not changed. We propose a model to explain these results and establish the genetic and biochemical basis of the camphor-0 phenotype.

## Materials and methods

### Plant material

Artemis is an F1 hybrid variety developed by Mediplant (Conthey, Switzerland), produced by crossing C4 and C1 parental material of East Asian origin (Delabays et al., 2001). Its artemisinin content has been reported to reach 1.4% of the leaf dry weight when grown in the field (Townsend et al., 2013). Artemis F1 population and M2 populations were created and grown from cuttings at Mediplant, Conthey, Switzerland and Yorkshire, UK field trial sites as described previously (Graham et al., 2010; Townsend et al., 2013). An F2 family-based pedigree population containing 662 individuals, created by selfing 84 randomly selected F1 Artemis individuals, was grown for 12 weeks in the glasshouse, under long- day conditions (16 hrs day/8 hrs night) at 22°C max/17°C min in P40 trays using Levington F2 seed and modular compost.

### Plant crossing

Plant crosses between camphor-0 M2 individuals grown from cuttings were created as described previously (Czechowski et al., 2016). Cuttings from parental genotypes were maintained in 10 cm diameter pots under 16-hour days for 12 weeks. Plants were then transferred to 12-hour days to induce flowering. Flowering was identified as the point at which the first ray florets were visible. Once flowering commenced, bags were placed over two plants to enable hybrid production. These bags were shaken every two days to encourage pollination. Once all flowers had died back the bags were removed and the flower heads allowed to dry out under glass for a further 6 weeks before harvesting.

### Metabolite analysis by Gas and Ultra-High Performance Liquid Chromatography – Mass Spectrometry

Metabolite analysis by GC- and UPLC-MS was performed as described previously (Czechowski et al., 2016). Eighteen plants from Artemis F1 and from progenies of one selected camphor-0 sibling cross were grown in glasshouse conditions from seeds in 10 cm diameter pots for 12 weeks under long- day conditions as described above. Metabolite profiles were generated from 50mg FW pooled samples of leaves at different developmental stages: 1-5 (counted from the apical meristem) representing the juvenile stage; leaves 7-9 representing the young, expanding stage; and leaves 11-13 representing the mature, expanded stage. Fresh leaf samples were stored at -80°C. Trichome-specific metabolites were extracted as described previously (Czechowski et al., 2016) with minor modifications. Briefly, 50 mg of fresh material was extracted by gentle shaking in 500 μl chloroform for 1 h.

For UPLC-MS analysis of sesquiterpenes, a diluted (1:5 (v/v) extract:ethanol) 2μL aliquot was injected on an Acquity UPLC system (Waters, Elstree, UK) fitted with a Luna 50×2 mm 2.5 μm HST column (Phenomenex, Macclesfield, UK). Metabolites were eluted at 0.6 mL/min and 40°C using a linear gradient from 40% to 100% acetonitrile containing 0.1% (v/v) formic acid over 2.5 min. Pseudomolecular [M+H]+ ions were detected using a Thermo Fisher LTQ-Orbitrap (ThermoFisher, Hemel Hempstead, UK) mass spectrometer fitted with an atmospheric pressure chemical ionization source operating in positive ionization mode under the control of Xcalibur 2.1 software. Data were acquired over the m/z range 100 - 1000 in FTMS centroid mode with resolution set to 7500 FWHM at m/z 400. Data extraction and analysis was performed using packages and custom scripts in R 3.2.2 (https://www.R-project.org/). XCMS (Smith et al., 2006) incorporating the centWave algorithm (Tautenhahn et al., 2008) was used for untargeted peak extraction. Deisotoping, fragment, and adduct removal were performed using CAMERA (Kuhl et al., 2012). Artemisinin was quantified using the standard curve of the response ratio of artemisinin (Sigma, Poole, UK) to internal standard (β-artemether; Hallochem Pharmaceutical, Hong Kong) added to extracts and standards. Metabolites were identified with reference to authentic standards or NMR-resolved structures and empirical mass formulae calculated using the R package rcdk (Guha, 2007) within 10 ppm error and elemental constraints of: C = 1–100, H = 1-200, O = 0–20, N = 0–1. Peak concentrations were calculated using bracketed response curves, where standard curves were run every ~30 samples. Metabolite concentrations were expressed as a proportion of the residual dry leaf material following extraction.

For analysis of monoterpenes and volatile sesquiterpenes, an aliquot of chloroform extract (prior to dilution with ethanol for UPLC analysis) was taken for GCMS analysis using an Agilent 6890 GC interfaced to a Leco Pegasus IV TOF MS (Leco, Stockport, UK). A 1μL aliquot was injected into a CIS4 injector (Gerstel, Mülheim an der Ruhr, Germany) fitted with a 2 mm ID glass liner containing deactivated glass wool at 10°C. The injector was ramped from 10°C to 300°C at 12°C/s then held at 300°C for 5 min. The carrier gas was He at constant flow of 1 mL/min and the injection split ratio was 1:10. Peaks were eluted using a Restek Rxi-5Sil MS column, 30m x 0.25 mm ID x 0.25 μm film thickness (Thames Restek, Saunderton, UK). The following temperature gradient was used: isothermal 40°C 2 min, ramp at 20°C/min to 320°C then hold for 1 min; total run time ~20 min. The transfer line was maintained at 250°C and the MS used to collect -70eV EI scans over the m/z range 20-450 at a scan rate of 20 spectra/second. Acquisition was controlled by ChromaTof 4.5 software (Leco). ChromaTof was used to identify peaks and deconvolute spectra from each run, assuming a peak width of 3s and a minimum s/n of 10. Peak areas were exported as deconvoluted total ion traces (DTIC) and annotated against authentic standards and NIST spectral matches. For semi-quantitative comparisons, DTIC peak areas were normalized to the added internal standard (tetradecane) and sample dry weight. A standard curve was created for camphor, to enable absolute concentration comparisons with artemisinin.

R stats base package, nlme, multcomp, and multcompView were used for all statistical data analysis

### Extraction and quantification of isoprenoid diphosphates (GPP, FPP, GGPP)

Extraction and quantification of isoprenoid diphosphates was performed as described previously (Catania et al., 2018). Twelve plants from Artemis F1 and from progenies of one selected camphor-0 M2 cross were grown in glasshouse conditions from seeds in 10 cm diameter pots for 12 weeks in a randomized way as described above. Juvenile leaves (leaf 1-5) were harvested from main stem and side branches and pooled from two plants to achieve around 1g of fresh material which was immediately flash frozen in LN2. The material was ground to a fine powder using a TissueLyser II ball mill fitted with stainless steel grinding jars (Qiagen, Crawley, UK) operated at 15 Hz for 15 sec with one repeat. Powdered leaf material was weighed out and extracted three times with 5ml of ice cold methanol:water (7:3, v/v), including a 0.3 µg/ml of each of three internal standards: geranyl-, farnesyl- and geranylgeranyl-S-thiolodiphosphates (GSPP, FSPP and GGSPP; Echelon Biosciences). Extracts were processed according to Nagel et al. (2014). Total extract volume was brought up to 20 ml with water. Briefly, each extract was passed through a Chromabond HX RA column (150 mg packing), which had first been conditioned with 5 ml methanol and 5 ml of water, and compounds eluted under gravity with 3 ml of 1 M ammonium formate in methanol. The eluate was evaporated under a stream of nitrogen to dryness, dissolved in 250 µL of water:methanol (1:1.v/v), and a 2 uL aliquot injected on a Waters Acquity I-Class UPLC system interfaced to a Thermo Orbitrap Fusion Tribrid mass spectrometer under Xcalibur 4.0 control. Isoprenoid compounds were eluted on a Waters Acquity C18 BEH column (2.1mm x 100 mm, 1.7 um) at 50°C using the following binary gradient program: solvent A = 20mM ammonium bicarbonate + 0.1% triethylamine; solvent B = 4:1 acetonitrile:water + 0.1% triethylamine; flowrate 0.4 ml/min; 0-100% B linear gradient over 4 minutes. Post column, compounds were ionized using a heated electrospray source (vaporizer = (250)°C; N2 flows for sheath/aux/sweep = 30/15/10 arbitrary units; source = 4kV; ion transfer tube = -30V and 275°C; tube lens = -40V). Data was acquired in full scan Ion trap mode with the following settings: 100-500 m/z range, max ion time 100ms, 1 microscan, AGC target = 3.00e+04.

No signal could be detected for GPP (elution time 2.1 min) or GGPP (elution time 3 min) in any of the biological samples analyzed, despite the clear signal observed for the 1-50uM linear GGPP/GGSPP response ratio calibration curve (R2 = 0.999) and for the 1-50uM linear GPP/GSPP response ratio calibration curve (R2 = 0.9913). FPP eluted at ~2.6 min and the internal standard (FSPP) at ~2.7 min. The deprotonated pseudomolecular ions ([M-H]-) of 381.1519 and 397.1261 for FPP and FSPP, respectively, were used for quantification (+/- 5ppm window) against a 1-100uM linear FPP/FSPP response ratio calibration curve (R2 = 0.9852), using Xcalibur 4.0 software (Thermo).

### RNA extraction, cDNA preparation and gene expression analysis using qRT-PCR.

Total RNA was extracted from the same leaf tissue as subjected to metabolite profiling analysis. Leaf tissue from juvenile expanding- and mature-stage leaves sampled as described above was ground to a fine powder using Qiagen Retsch MM300 TissueLyser (Qiagen, Hilden, Germany) and total RNA extracted using the RNAeasy kit with on-column DNaseI digestion step (Qiagen, Hilden, Germany). RNA was quantified using NanoDrop-1000 (NanoDrop products, Wilmington, USA) and its integrity was checked on agarose gels. 2 ug of total RNA was reversely transcribed using SuperScript II kit (Life Technologies Ltd, Paisley, UK) and Oligo(dT)12-18 Primer (Life Technologies Ltd, Paisley, UK) according to manufacturer’s instructions. Expression levels of putative *Bornyl diPhosphate Synthase (AaBPS)* and its two close homologues: *AaBPS-likeA* and *AaBPS-likeB; Farnesyl diPhosphate Synthase (FPS), amorpha-4,11-diene synthase (AMS)*, *amorpha-4,11-diene C-12 oxidase (CYP71AV1)*, *cytochrome P450 reductase (CPR), artemisinic aldehyde Δ 11 (13) reductase (DBR2)* and aldehyde dehydrogenase *(ALDH1)*, relative to *ubiquitin (UBQ)* were determined by qPCR as described before (Czechowski et al., 2018). Reactions were run in 3 technical replicates. Gene-specific primers used are detailed in **Table S3**. Real-time PCR was performed on CFX384 Teal-Rime System (Bio-Rad Laboratories) using SsoAdvanced Universal SYBR^®^ Green Supermix (Bio-Rad Laboratories). Each 10-μL reaction contained 1 μL of a 5-fold dilution of the cDNA synthesis reaction, 5μL of 2X supermix, and primers at a final concentration of 250 nM. The cycling conditions included an initial activation step for 30 s at 98°C followed by 40 cycles of denaturation at 98°C for 10 s and annealing/extension at 60°C for 30 s. Fluorescence data were acquired during the annealing/extension phase. A melt curve was obtained at the end of the amplification to allow confirmation of product specificity. C_T_ values were obtained using CFX Manager Software (Bio-Rad laboratories) and amplification efficiencies (E) obtained using LinReg PCR (Ruijter et al., 2009). Transcript abundance for the gene of interest (GOI) relative to *UBiQuitin* gnene (UBQ) was determined using the formula: $GOI\text{ expression }level={\left(E_{GOI}\right)}_{T}^{\Delta C}/{\left(E_{UBQ}\right)}_{T}^{\Delta C}$ .

### Genomic DNA extractions

For DNA extraction 30-50 mg of fresh leaf material was harvested from plants growing in the glasshouse. DNA was extracted using Qiagen BioSprint 96. Extracted DNA was quantified spectrophotometrically using NanoDrop-8000 (NanoDrop products, Wilmington, USA) and normalized to 10 ng/ul for genotyping assays, inverse PCR and other PCR analysis.

### Genotyping analysis of camphor-0 and Artemis F2 Populations.

Allele specific primers for *AaBPS, AaBPS-likeA and AaBPS-likeB* genomic DNA sequences were designed for KASPar and ABI3730xl genotyping assays based on the regions allowing to distinguish between closely related sequences, as depicted on **Figure S5A**. Primer sequences are listed in **Table S3**.

Twenty nanograms of leaf genomic DNA extracted from individual Artemis F2 plants was used for 10ul KASPar assay reaction containing: 1x KASP V4.0 low ROX master mix (LGC Genomics, Teddington, UK); 167nM of each of the two allele specific primers and 414nM of universal primer according to the manufacturer’s recommendations. Allelic discrimination runs and allelic discrimination analysis were performed on Viia7 system (Life Technologies Ltd, Paisley, UK) according to manufacturer’s recommendations.

For the ABI3730xl SNP assays, two differentially sized primers specific to each SNP alleles were designed and used in one PCR reaction with a common, locus specific primer containing M13 tail. A mismatch base at position -4 or -5 from 3’ end of each allele-specific primer was introduced to increase allele-specificity of the PCR reactions. Universal fluorescent (FAM) labeled M13 primers were included in the reaction to incorporate FAM dye label to allow visualisation on the capillary apparatus. PCR amplification was performed in 10 μl total volume, with 2 ng genomic DNA, 1x AmpliTaq Gold^®^ PCR Master Mix (Applied Biosystems, Foster City, CA) containing 0.25 Units of AmpliTaq Gold, 50 nM forward and reverse primers and 750nM M13 primer. PCR was carried out with 40 cycles using an annealing temperature of 60°C. PCR reactions were diluted 1:20 in H2O and fractionated on an ABI 3730xl capillary sequencer (Applied Biosystems, Foster City, CA). SNPs were analyzed and scored using GeneMarker™ software (Softgenetics, State College, PA).

### Inverse PCR of *AaBPS* 5’ flanking region

Inverse PCR was carried out on genomic DNA essentially according to the method of Ochman et al. (1988) except that a second round of PCR was included giving a linear product containing DNA flanking the BPS gene. Nested primers were designed around the AaBPS sequence as follows: primer pair 1 (outer) comprised BPS5’_F1 and BPS5’_R1; primer pair 2 (inner) comprised BPS5’_F2 and BPS5’_R2. (Primer sequences are listed in **Table S3**). 250 ng of genomic DNA extracted from C1 and C4 Artemis parents was digested with *BalI* and diluted 1:10, 1:100 and 1:1000. Inverse PCR was carried out using QIAGEN Multiplex PCR Kit (Qiagen, Crawley, UK) with primer pair 1 (outer) with dilutions of *BalI-*digest and PCR conditions were: 95°C for 15min followed by 40 cycles of denaturation at 95°C for 30 s, annealing at 54°C for 30 s and extension at 72°C for 5 min, which was followed by final extension at 72°C for 5 min. Nested PCR was carried out on 1 ul of undiluted inverse PCR products using inner primer pairs for 5’-flanking ends, QIAGEN Multiplex PCR Kit (Qiagen, Crawley, UK) and the same cycling conditions as above except annealing was carried out at 61°C. Nested PCR resulted in 1934bp product which was gel-purified, diluted 1/10 and ligated into Strataclone vector using Strataclone PCR cloning kit (Beckman Coulter Genomics, Takeley, UK) and StrataClone solo competent cells transformed by heat shock. Positive clones were sent for Sanger sequencing using M13 universal primers.

### Construction of *AaBPS::Gusi* vector and *A. annua* transformations

The pSAT7a vector (Tzfira et al., 2005) was used to create the *AaBPS promoter::Gusi* reporter fusion construct. The Gusi sequence was extracted from the *pBI121::Gusi* using the Sac I and Sal I restriction sites and ligated into the *pSAT7a* vector to create a *pSAT7a::Gusi* vector. A 1934bp fragment of the BPS promoter was amplified from genomic DNA extracted from Artemis parent C1, using the primers tailed with *AgeI* and *NotI* restriction sites, listed in **Table S3**. Restriction digest was carried out following PCR and the digested amplified fragments cloned and verified by sequencing prior to cloning into the *pSAT7a::Gusi* vector. The full *AaBPS promoter::Gusi* construct (3357bp) was digested out of the *pSAT7a* using *AgeI* and *NotI* restriction sites and the fragment blunted using T4 DNA polymerase. The construct was cloned into the *pRSC2* binary vector using the *EcoRV* site and then the resulting colonies were verified for orientation and sequence prior to transformation into the binary vector pRSC2. The binary vector was then transformed into *Agrobacterium tumefaciens* (LBA4404) by electroporation and 100 µl glycerol stocks set up for subsequent plant transformations. Transformation of *Artemisia annua* Artemis was carried out following the protocol described by Catania et al. (2018).

### Histochemical Gus staining

Gus (β-glucoronidase) staining of transformed material was carried out following the protocol described by Jefferson et al. (1987). Briefly plant material for staining was submerged in GUS stain and vacuum infiltrated for 20 minutes followed by incubation at 37°C. Samples were incubated for up to 24 hours. The reaction was followed by observation with a dissecting microscope and stopped when the stain was sufficiently developed. To enable the stain to be more clearly visualized the samples were cleared with successive washes in 70% ethanol at 37°C.

### PCR analysis of *AaBPS* locus

Primers covering the entire AaBPS genomic sequence with 5’ and 3’ flanking sequences obtained from inverse PCR were designed as depicted on **Figure 5A**. Primer sequences are listed in **Table S3**. Genomic DNA was extracted from glasshouse grown fourteen camphor-0 M2 lines, two randomly selected Artemis F1 individuals and from C1 and C4 Artemis parents. Twenty nanograms of genomic DNA was used in 20 ul PCR reactions containing 500nM of each forward and reverse primer, 1U of Phusion^®^ High-Fidelity DNA Polymerase NEB, and 200nM of dNTPs. PCR conditions were as follows 98°C - 30 sec, followed by 10 cycles of 98°C – 10 sec 70°C – 30sec Touch down) – decrease 1°C per cycle, 72°C – 2.5 min, followed by 30 cycles of 98°C – 10 sec, 60°C – 30 sec, 72°C – 2.5 min and final extension at 72°C for 5 min. PCR products were ligated into Strataclone vector using Strataclone PCR cloning kit (Beckman Coulter Genomics, Takeley, UK) and StrataClone solo competent cells transformed by heat shock. Positive clones were sent for Sanger sequencing using M13 universal primers.

### Heterologous expression of AaBPS and purification of recombinant protein

In order to confirm the catalytic function of AaBPS, we carried out heterologous expression in BL21 (DE3) *E. coli* strain. ChloroP analysis revealed that has a putative plastid targeting (PT) sequence at the 5’ end. Three sequences were tested for expression: full length and two truncated forms. ChloroP and TargetP analysis predicted the PT sequence cleavage site after residue A34 in the predicted amino acid sequence. BPS_tr1 was truncated to this point, ie: sequence begins at residue C35. Whittington et al. (2002) reported a longer N-terminal PT region in Sage BPS, beginning the coding region just before the active site lid residues. BPS_tr2 begins at R58, the start of the predicted active site lid residues of *Aa*BPS. Primers for PCR cloning of the AaBPS sequence were designed to incorporate either an NheI site at the 5’ end of the coding sequence, and a BamHI site at the 3’ end. After sequencing to confirm accuracy, products were cloned into either the NheI-BamHI or sites of pET28a, as appropriate and then sub-cloned into pDONR207 entry vector. The three AaBPS versions (Full length and two truncations) were then transferred into pH9GW destination vector *via* the LR reaction and then transformed into BL21 (DE3) *E. coli* strain for protein expression. Expression was scaled up to 1 L cultures and protein purified using 2‐step large scale purification processes using metal affinity chromatography (1 ml) coupled to a Superdex 16/600 200pg gel filtration column (120 ml). Between 50 and 300 μg of each concentrated protein was obtained using this purification method for the subsequent activity assays.

### AaBPS activity assay

We adapted an activity assay that had been used in the lab to assay a prenyl‐diphosphate synthase together with an assay used for the Sage bornyl diphosphate synthase (Wise et al., 1998). The reaction contained: 1X MTC buffer, 1mM DTT, 10mM MgCl_2_, and 75μM GPP were set up in 2mL glass vial in total volume of 500μL. Reactions were started by addition of 50 μl protein (0.15 or 0.85 μM final concentration) and overlaid with 500 μl pentane and incubated at 31°C for 3 hours with slow shaking (130 rpm). To hydrolyse the pyrophosphate product, 20 units of rAPid alkaline phosphatase (Roche 04898133001) was added to the aqueous layer and gently mixed. Reactions were incubated at 30°C for 2 hours. Vials were vortexed vigorously for 10 seconds, then centrifuged at 1,500 xg for 10 minutes. 2μl of the organic layer was sampled directly out of the vial and injected onto the GC‐MS following the GC‐MS method used before (Wise et al., 1998).

## Results

### A single recessive allele is responsible for camphor-0 phenotype in Artemis M2 and F2 plants

**Figure 1:**
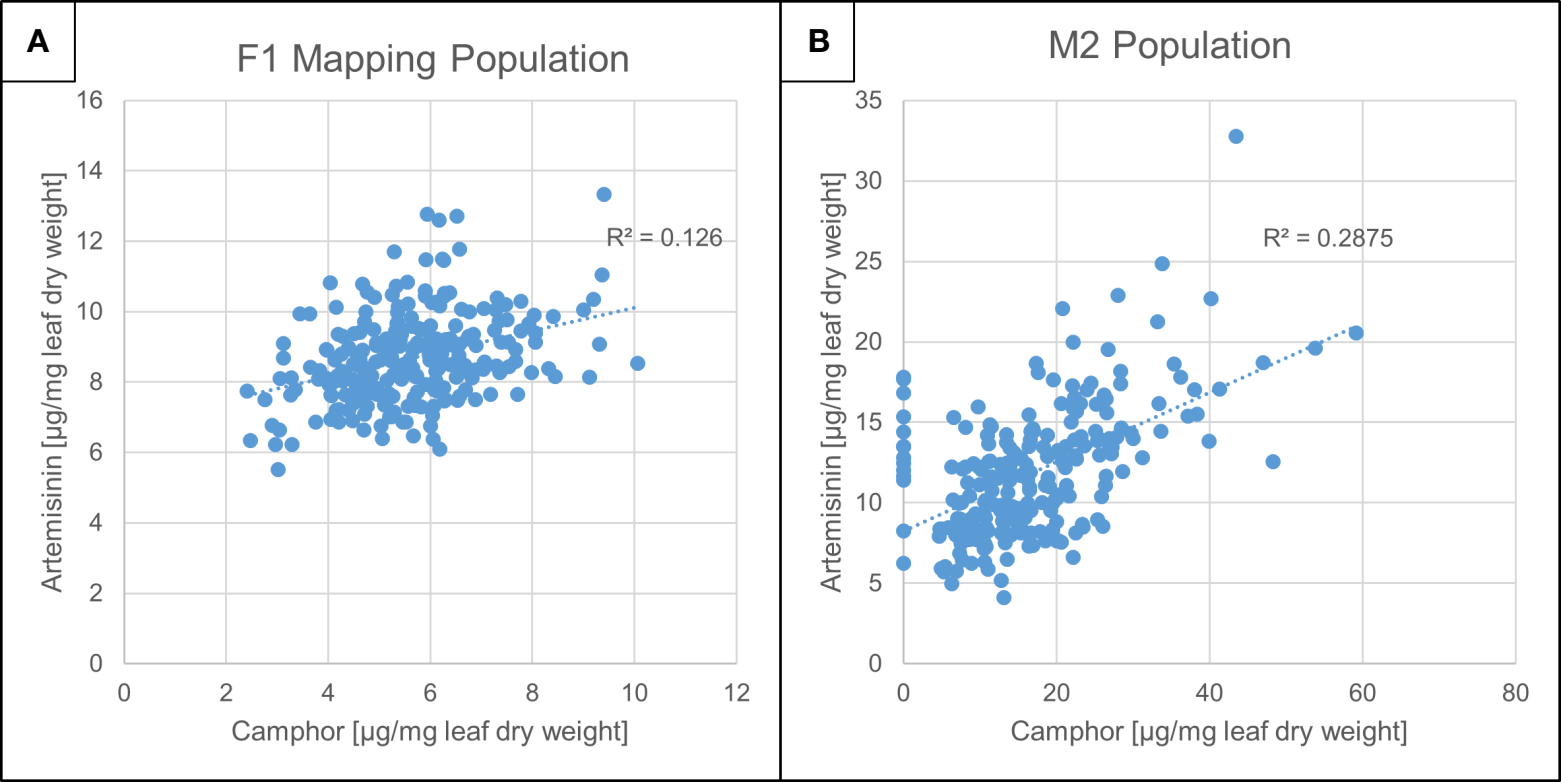
Artemisinin and camphor concentration in *Artemis* F1 mapping- and M2 mutant populations. Camphor and artemisinin concentration in dry leaf material from Artemis F1s **(A)** and M2 **(B)** populations grown in Mediplant (Switzerland) field trials (2008). Leaf material was harvested from 248 F1 and 233 M2 individuals, extracted and analyzed by ultra-high performance liquid- and gas chromatography–mass spectrometry (UPLC- and GC-MS) as described in Materials and Methods. Camphor and artemisinin levels were quantified against standard curves using authentic standards.

We previously performed EMS mutagenesis on the *A. annua* Artemis F1 hybrid and produced an M2 population (Graham et al., 2010; Czechowski et al., 2016). This population was grown in parallel with an F1 Artemis mapping population in multiple field trials in the UK and Switzerland and individuals from each were subjected to phenotyping that included detailed metabolite profiling (Larson et al., 2013; Townsend et al., 2013). Concentrations of artemisinin and camphor in dry leaf material from these two field grown populations were quantified against standard curves as described in Materials and Methods (**Figure 1**). The concentration of camphor on a leaf dry weight basis (maximum of 1%) was of a similar order to artemisinin (maximum of 1.4%) in the Artemis F1 field grown material (**Figure 1A**). We performed a glasshouse based screen to select about 10% of plants from the M2 population on the basis of high artemisinin content as previously described (Graham et al., 2010; Larson et al., 2013; Townsend et al., 2013). The vast majority of these individuals showed elevated camphor and artemisinin content reaching 6% and 3% of leaf dry weight, respectively, with camphor concentration actually exceeding that of artemisinin in most of the lines (**Figure 1B**). That camphor and artemisinin concentrations show a strong positive correlation in individuals from both F1 and M2 populations (**Figure 1A, B**) suggested that the monoterpene and sesquiterpene pathways are not competing for flux from the plastidial isoprenoid pathway (Schramek et al., 2010). The analysis revealed that 14 out of the 233 M2 field-grown lines almost completely lacked camphor but had relatively normal levels of artemisinin (**Figure 1B**) and no morphological alterations (data not shown). All 14 of these camphor-0 M2 lines came from different M1 parents, suggesting that some form of segregation rather than mutagenesis may be responsible for emergence of the camphor-0 phenotype in the M2 material. The Artemis F1 hybrid variety is derived from a cross between two heterozygous parents (Delabays et al., 2001; Graham et al., 2010). We selfed 85 randomly chosen Artemis F1 individuals that had not been subjected to EMS mutagenesis and measured camphor and artemisinin in dry leaves from 662 of the resulting F2 progeny and found that 126 of these exhibited the camphor-0 phenotype (**Figure S1**). That the camphor-0 phenotype is not present in F1 plants but appears in F2 populations (**Figure 1** and **Figure S1**) strongly suggests that the Camphor-0 phenotype is not due to EMS mutagenesis but is instead due to segregation of a recessive trait that emerges in the F2 generation having been acquired from one or other of the parental lines which we previously showed contain a high level of heterozygosity (Graham et al., 2010).

We performed test crosses on five of the selected camphor-0 M2 individuals that confirmed the phenotype was due to a single genetic locus as described in Materials and Methods (**Figure S2** **).** Progeny of these crosses were all camphor-0 (**Figure S2**) further confirming the phenotype being due to a single recessive allele that had also been fixed in the M2 material. Camphor-0 progeny of test-crosses displayed the same morphology as Artemis F1 controls when grown under glasshouse conditions (**Figure S3**).

### Metabolite and gene expression profiling of developmental stages of camphor-0 leaves

**Figure 2:**
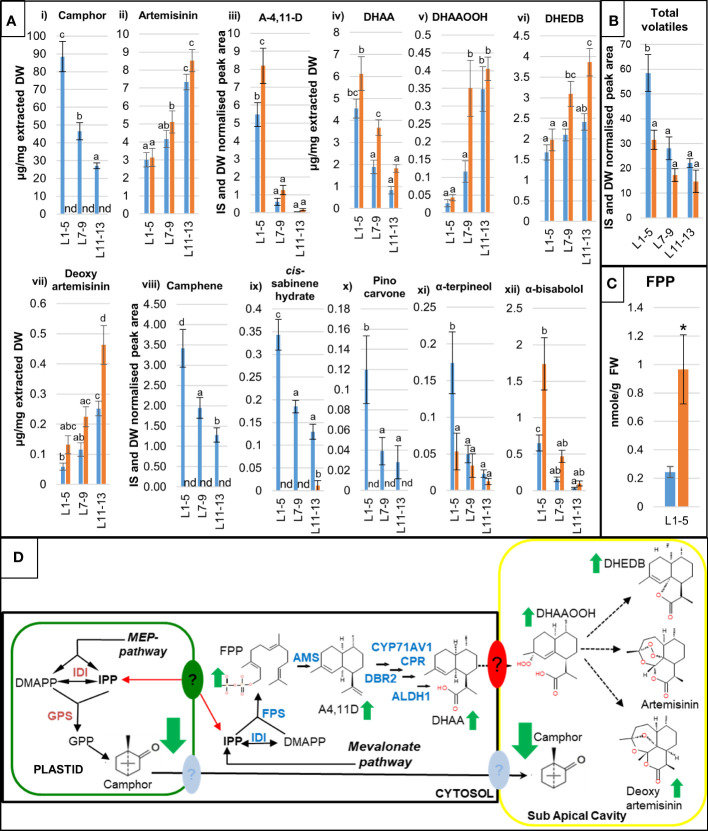
Detailed molecular characterization of camphor-0 M3 lines. Levels of artemisinin-related and other selected mono- and sesquiterpenes **(A)** were quantified by gas chromatography–mass spectrometry (GC-MS) **(i)**, (iii), (viii) - (xii) and ultra-high performance liquid chromatography–mass spectrometry (UPLC-MS) (ii), (iv)-(vii) analysis of extracts from fresh leaves L1-5 (juvenile), L7-9 (expanding), L11-13 (mature) as counted from the apical meristem from 12-weeks old glasshouse-grown Artemis F1 (blue bars) and selected camphor-0 M3 (orange bars); error bars – SE (n=5). nd - not detectable. GC-MS data for camphor were converted from internal standard (IS) and dry weight (DW) normalised peak areas to µg/mg extracted dry weight (vii) against a standard curve run with pure camphor. For both **(A, B)** letters represent Tukey’s range test results after one way ANOVA or REML. Groups not sharing letters indicate statistically significant differences. **(B)** Level of total volatiles measured by GC-MS in three types of leaf material as indicated in **(A)**. **(C)** Level of FPP measured in extracts from fresh juvenile leaves L1-5 Artemis F1 (blue bars) and selected camphor-0 M3 (orange bars); FW - fresh weight, error bars – SE (n=3), *- statistically significant difference (t-test) between Artemis and camphor-0 at p<0.05. **(D)** Summary of metabolite changes in the camphor-0 glandular secretory trichomes (GSTs), full arrows – known enzymatic steps, dotted arrows – potential non enzymatic conversions, full green arrows – metabolite changes (all leaf stages). Metabolite abbreviations: GPP - geranyl diphosphate, FPP – farnesyl diphosphate, A-4,11-D – amorpha-4,11-diene, DHAA - dihydroartemsinic acid, DHAAOOH- dihydroartemisinic acid tertiary hydroperoxide, DHEDB – dihydro-epi-deoxyarteanniun B, Enzyme abbreviations: IDI - Isopentenyl Diphosphate Isomerase, GPS - Geranyl diPhosphate synthase, BPS - Bornyl diPhosphate synthase, FPS- Farnesyl diPhosphate Synthase. Artemisinin pathway: AMS – amorpha-4,11-diene synthase, CYP71AV1 - amorpha-4,11-diene C-12 oxidase, CPR – cytochrome P450 reductase, DBR2 - artemisinic aldehyde Δ 11 (13) reductase, ALDH1 - aldehyde dehydrogenase. Question marks indicate putative active transport systems operating in *A annua* GSTs.

Three distinct leaf developmental stages: young (leaves 1-5), expanding (leaves 7-9) and mature (leaves 11-13) were harvested from individual plants and subjected to metabolite (GC- and UPLC-MS) and gene expression (qRT-PCR) profiling as described in the Materials and Methods. This developmental series captures the major transition points in artemisinin biosynthesis and wider terpenoid metabolism in leaves of *A. annua* (Czechowski et al., 2016; Czechowski et al., 2018). The metabolite analysis revealed that camphor is the most abundant terpenoid detected in Artemis F1 leaf extracts with concentration reaching up to 10% of extracted dry weight in young leaves from glasshouse grown material (**Figure 2A** **i)).** While artemisinin levels remained unaltered in camphor-0 material (**Figure 2A** **ii),** **Table S1**), other significant changes in artemisinin-pathway metabolites were detected including an increase in the artemisinin precursors amorpha-4,11-diene (A-4,11-D) and dihydroartemisinic acid (DHAA) in young and expanding leaves (**Figure 2A** **iii**) and iv),**Table S1** and **Table S2**). There was also a significant increase in the level of dihydroartemisinic acid tertiary hydroperoxide (DHAAOOH), a previously described intermediate of non-enzymatic conversion of DHAA, in camphor-0 expanding leaves (**Figure 2A** **v**) and **Table S1**). We also observed elevated levels of two products of the alternative non-enzymatic conversion of DHAA: deoxyartemisinin and dihydro-epi-deoxyarteannuin B (DHEDB) in mature leaves of camphor-0 lines, when compared with Artemis F1 (**Figure 2A** **vi**) and vii), **Table S1**). The significant increases in artemisinin precursor metabolites in camphor-0 material were not accompanied by changes in the level of transcripts of Artemisinin-pathway genes, except for a marked increase in *AaDBR2* transcript in camphor-0 mature leaves (**Figure S4**).

Other, less abundant monoterpenes were also missing (camphene, *cis-* and *trans-*sabinene hydrate, pinocarvone, carvone) or strongly reduced (α-pinene, α-terpineol) in all leaf types of camphor-0 lines. (**Figure 2A** **viii**) – xi) and **Table S2**). Monoterpenes missing in the camphor-0 lines represent around 50% of the total volatiles measured by GC-MS in Artemis F1 young leaves (**Table S2**), which is reflected by almost a 2-fold reduction of total volatile content of camphor-0 young leaves (**Figure 2B**). There was, however, a significant increase in some sesquiterpenes such as α-bisabolol and spathulol (**Figure 2A** **xii**) and **Table S2**), in addition to the artemisinin-pathway metabolites detailed above. To further investigate the increase in sesquiterpenes unrelated to the artemisinin pathway in camphor-0 material, we measured the level of the isoprenoid precursors GPP, FPP and GGPP in juvenile leaf material using previously described protocols (Nagel et al., 2014; Catania et al., 2018). While GPP and GGPP were undetectable in all extracts, FPP levels were elevated by approximately 5-fold in camphor-0 juvenile leaves compared to F1 Artemis (**Figure 2C**). FPP synthase gene transcript levels were unchanged in camphor-0 material (**Figure S4**).

Metabolite changes in the camphor-0 glandular secretory trichomes (GSTs) are summarized in **Figure 2D**.

### Absence of *AaBPS* from the *A. annua* genome correlates with the camphor-0 phenotype

**Figure 3:**
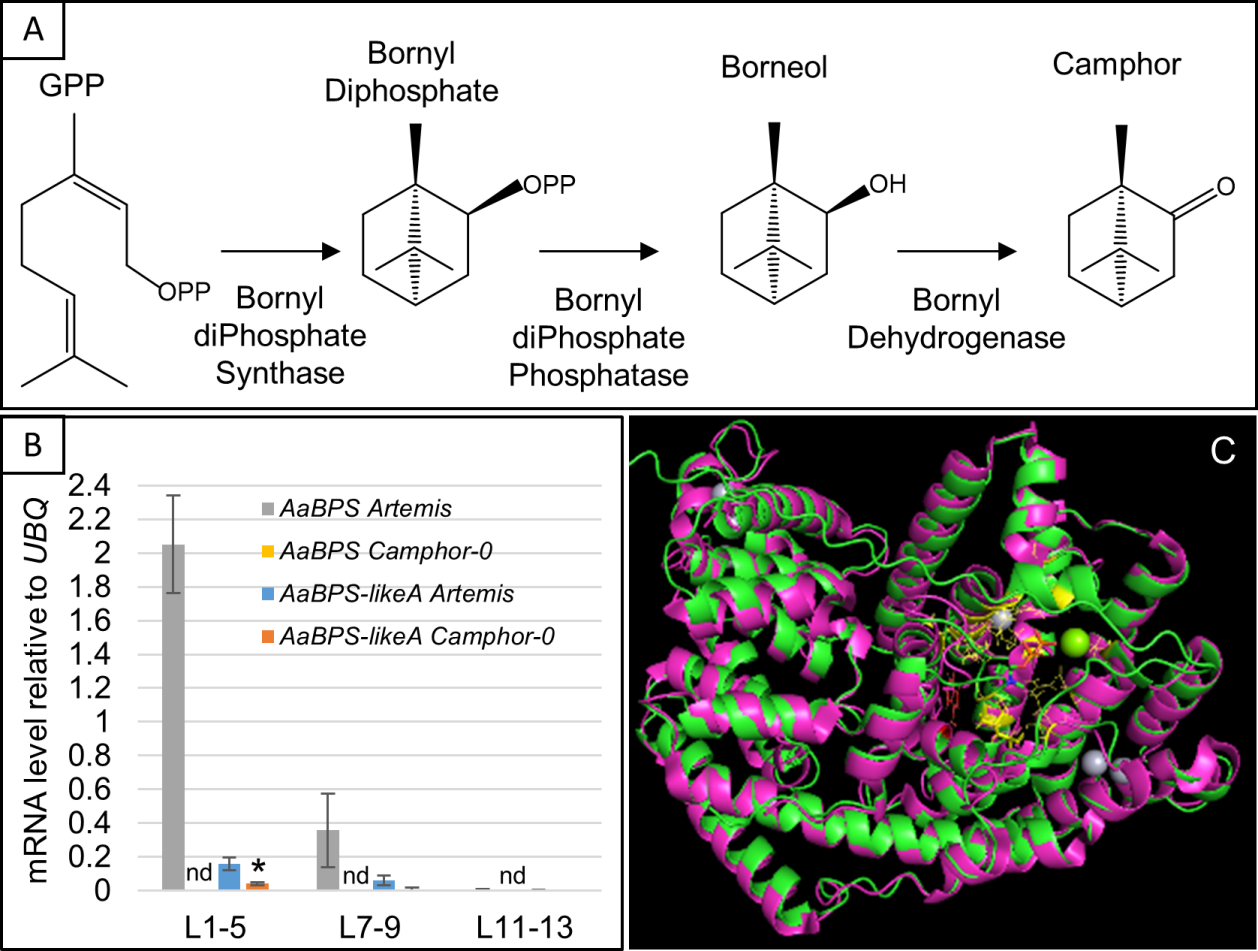
Identification of the *AaBPS* coding sequence and *AaBPS* expression. **(A)** Camphor biosynthesis pathway modified from (Croteau et al., 1981). **(B)** Expression of *AaBPS* and *AaBPS-likeA* in L1-5 juvenile, L7-9 expanding and L11-13 mature leaves. Error bars – SE (n=12). nd- transcript not detectable by qRT-PCR. * - statistically significant difference (t-test) between Artemis and camphor-0 at p<0.05. **(C)** Predicted AaBPS protein sequence was modelled (green) using the I-TASSER approach (C-score 0.5) and overlaid on sage (purple) Bornyl-diPhosphate Synthase (SoBPS, pdb structure 1N1B, 40% amino acid identity) I-TASSER predicted GPP binding residues highlighted in yellow, predicted catalytic W326 in red. Mg^2+^ (green) and Hg^2+^ (grey) ions from 1N1B structure are also shown.

BPS catalyses the first committed step in camphor biosynthesis (**Figure 3A**). BLAST analysis of an *A. annua* EST library (Graham et al., 2010) had previously revealed several monoterpene synthases that had been functionally characterized as linalool synthases *QH1* and *QH5 (* Jia et al., 1999 *)* and β-pinene synthase *QH6* (Lu et al., 2002). One candidate monoterpene synthase was designated *BORNYL-DIPHOSPHATE SYNTHASE* (*AaBPS*) based on highest sequence homology to other characterized plant *BPS* genes. The predicted protein sequence of the *AaBPS* contains a putative plastid targeting sequence at the 5’ terminus. *AaBPS* was found to be preferentially expressed in trichomes or trichome-containing tissues of *A. annua* at levels higher than any of the other monoterpene synthase candidates (Graham et al., 2010), and was the only gene from the camphor biosynthetic pathway, annotated in *A. annua* EST library (Graham et al., 2010), therefore a plausible candidate to investigate further.

We also identified two other *AaBPS-like* cDNA sequences that we designated as *AaBPS-likeA* and *AaBPS-likeB*. These both have 93% nucleotide identity with *AaBPS* and predicted amino acid identities of 87% for *AaBPS-likeA* and 88% for *AaBPS-likeB*. However, these sequences differ from *AaBPS* at conserved positions in the active- and substrate binding sites (**Figure S5A**). The low degree of nucleotide variation between these three genes led us to develop three gene specific SNP-based molecular markers which we used to genotype camphor-0 and camphor-containing material using KASPar and ABI3730 platforms as described in Materials and Methods (**Figure S5B**). qRT-PCR gene expression analysis of mRNA isolated from the three leaf developmental stages revealed that, while *AaBPS* is expressed at high levels in young leaves and lower levels in expanding leaves of Artemis F1 (**Figure 3B**), it is not detected in mRNA from any of the three leaf stages of camphor-0 plants (**Figure 3B**). *AaBPS* gene expression across the different leaf stages of Artemis F1 (**Figure 3B**) correlates with camphor levels (**Figure 2A** **i)** and is similar to a number of genes involved in artemisinin biosynthesis including *Amorpha-4,11–diene synthase* (*AaAMS*), *Amorpha-4,11–diene C12 oxidase (AaCYP71AV1), Artemisinic aldehyde Δ11(13) reductase* (*AaDBR2)* and *Aldehyde dehydrogenase (AaALDH1)* (**Figure S4**). *AaBPS-likeA* expression follows a similar pattern of expression to *AaBPS*, but at 10-fold lower levels in both Artemis F1 camphor-containing and camphor-0 material (**Figure 3B**). *AaBPS-likeB* transcripts were not detected in any of the leaf material analyzed by qRT-PCR.

We used the Iterative Threading ASSEmbly Refinement (I-TASSER) approach (Yang and Zhang, 2015; Zhang et al., 2017) to perform protein structure predictions on amino acid sequences of *AaBPS*, *AaBPS-likeA* and *AaBPS-likeB*. While the predicted AaBPS protein structure overlaid very well with the bornyl diphosphate synthase crystal structure from *Salvia officinalis* (SoBPS, **Figure 3C**) the AaBPS-likeA and AaBPS-likeB overlays with SoBPS were both disrupted at the conserved GPP binding site which would appear to be due to the presence of phenylalanine at position 346 rather than leucine at position 347 and isoleucine at position 343 in AaBPS and SoBPS, respectively (**Figures 3C**, **S5C**). The SoBPS active site cavity is considered to be a tight fit for the GPP hydrocarbon chain with an estimated packing density of the enzyme–substrate complex of around 78% (Whittington et al., 2002). The presence of a large aromatic amino acid, such as phenylalanine at the GPP binding site of AaBPS-likeA and AaBPS-likeB could result in steric hindrance and disrupt any monoterpene synthase function of these AaBPS homologues (**Figure S5C**).

### The *AaBPS* promoter drives high reporter gene expression in glandular secretory trichomes and hair-like (T-shape) non-secretory trichomes

**Figure 4:**
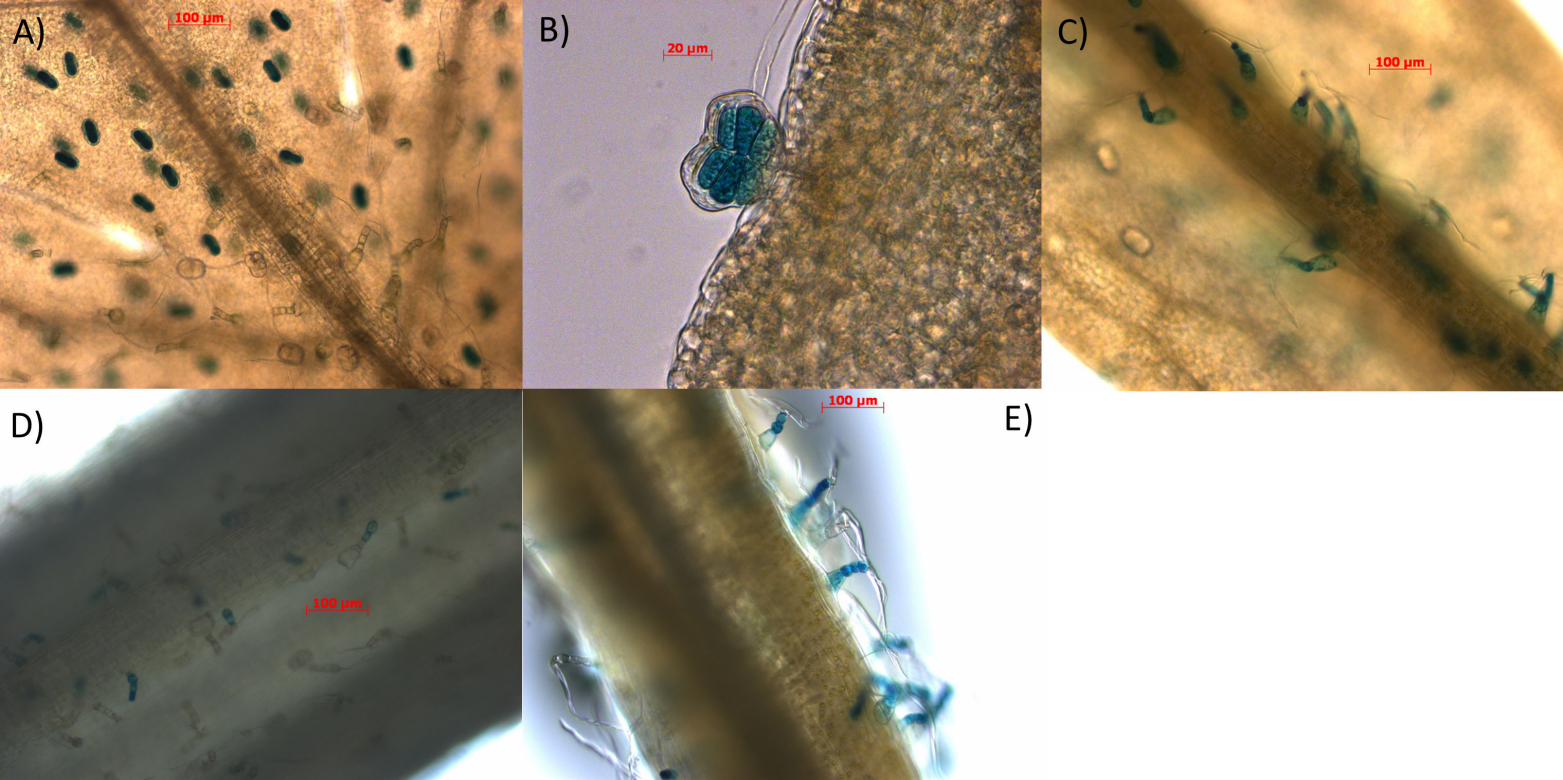
Characterisation of *AaBPS* promoter using promoter-GUS fusions. Tissues of 12-weeks old transgenic lines expressing *pAaBPS::GUSi*: **(A)** juvenile leaf (10x) **(B)** juvenile leaf (40x) **(C)** mature leaf (10x) **(D)** leaf petiole (10x) **(E)** stem (10x) were stained for *GUS* activity as described in Materials and Methods and photographed using bright-field microscopy (magnification indicated in the brackets). Scale bars indicated for each picture in red.

Inverse PCR was performed on genomic DNA isolated from Artemis F1 material and the resulting 1934bp sequence upstream of the *AaBPS* start codon was cloned upstream of the *beta-glucuronidase* (*GUS*) reporter gene and the resulting construct was used to transform Artemis F1 material, using a previously described *Agrobacterium tumefaciens* based protocol (Catania et al., 2018) as described inMaterials and Methods). GUS activity staining of various tissues from the T2 generation of *promBPS::GUSi* transformed plants showed that the *AaBPS* promoter drives high GUS expression in glandular secretory trichomes of the juvenile leaves (L1-5) and hair-like (T-shape) non-secretory trichomes present in mature leaves (L11-13), leaf petioles and stems (**Figure 4**).

### Camphor-0 lines lack the entire AaBPS gene locus

**Figure 5:**
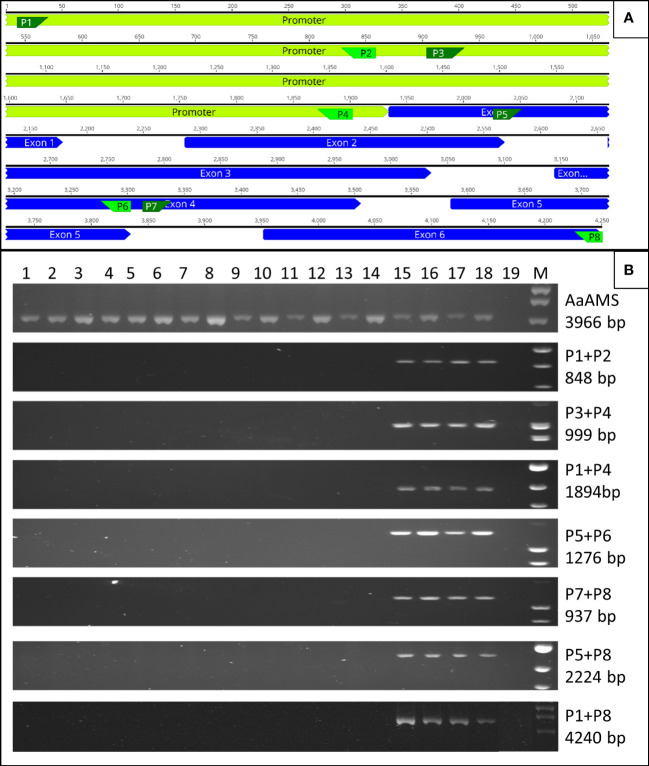
Genomic deletion in camphor-0 lines. **(A)** Location of primers (P1-P8) designed for various parts of *AaBPS* gene including5’ promoter region (Primer sequences in **Table S3**). **(B)** PCR amplification on genomic DNA isolated from 14 camphor-0 M2 (lines 1-14), two individual Artemis F1 (lines 15-16), Artemis Parents C4 (line 17) and C1 (line 18) using primers annotated on panel **(A)** Line 19 – no template control. Line M – Gene Ruler™ 1kb DNA ladder (Thermo Fisher). Amplification of full length *AaAMS* gene (GenBank Accession AF327527) used as a positive control. Size of PCR amplicons predicted from genomic DNA sequence shown.

To further investigate the *AaBPS* locus in camphor-0 material we used Artemis F1 genomic DNA to PCR amplify a 4.25 kb region that included promoter sequence obtained by inverse PCR and confirmed by *promoter*::*GUS* fusions (**Figure 4**). Primer pairs were then designed across the 4.25 kb region (**Figure 5A**) and used to establish that the *AaBPS* sequence was absent from genomic DNA of 14 camphor-0 M2 individuals but present in genomic DNA from Artemis F1 and the Artemis C1 and C4 parents (**Figure 5B** **).** Genomic DNA from camphor-0 M2 individuals did amplify a 3.97 kb fragment containing *Amorpha 4,11 diene synthase* gene (**Figure 5B**). *AaBPS* locus-related PCR products were cloned and verified by DNA sequencing. While this PCR analysis does not define the entirety of the genomic deletion in camphor-0 plants, our analysis does show that both the promoter and the entire coding region of the *AaBPS locus* are missing.

### Recombinant AaBPS protein performs the first committed step in camphor biosynthesis

**Figure 6:**
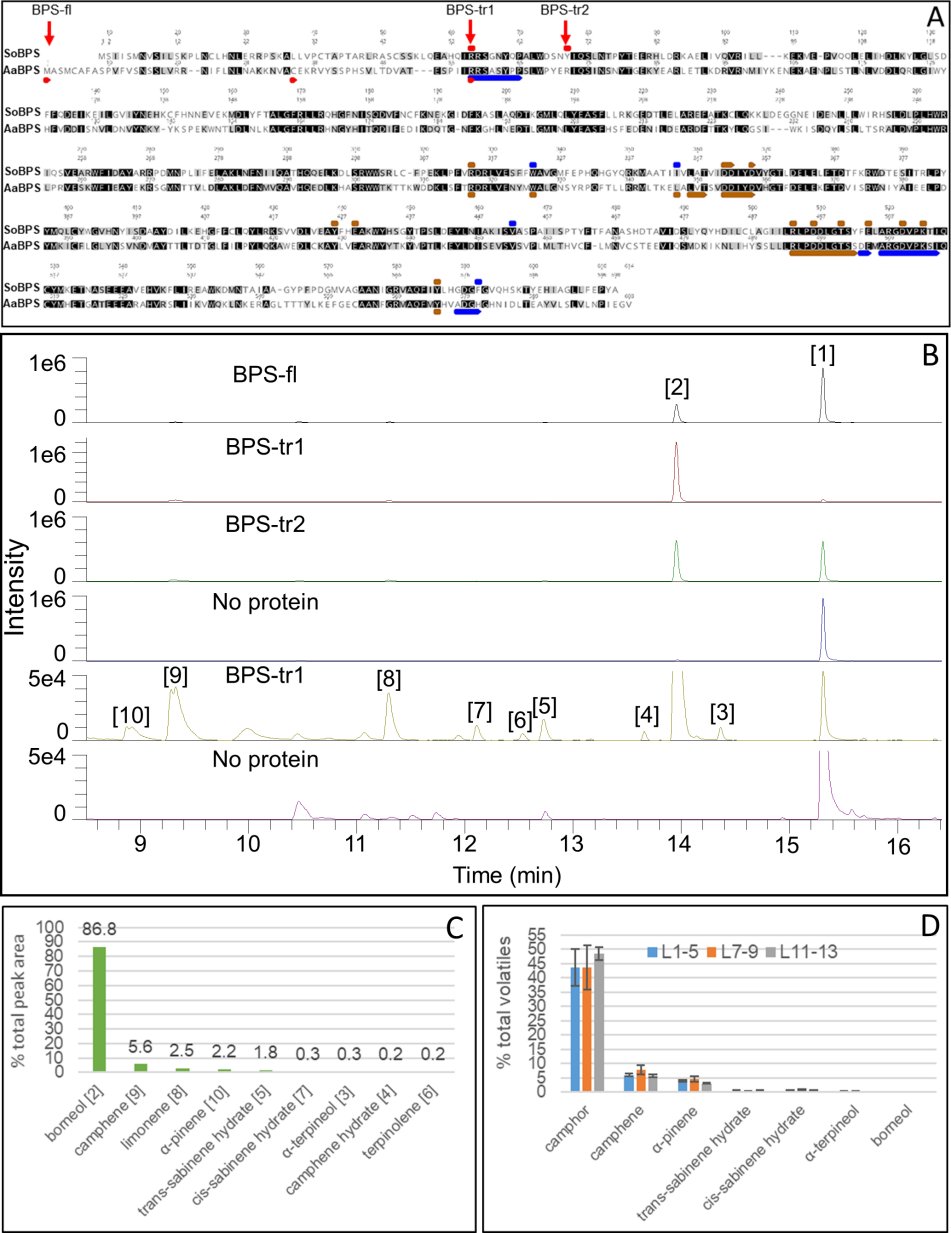
Functional characterisation of *AaBPS*. **(A)** Alignment of predicted amino acid sequence of AaBPS (GenBank accession OL656813) with SoBPS (GenBank accession AF051900). Truncation points used to generate truncated versions of AaBPS are indicated as BPS-tr1 and BPS-tr2. BPS-fl represents a full length protein. Red bars – active site lid, Blue bars - active site, Brown bar – substrate binding site annotated from Whittington et al., 2002. Identical (Black) and similar (grey) positions highlighted. **(B)** AaBPS *in vitro* protein activity assay using gas chromatography–mass spectrometry (GC-MS) based detection of monoterpenes. No protein control includes geraniol, the product of GPP hydrolysis (peak 1). Identification of peak 1 and 2 as geraniol and borneol respectively was assigned using known standards. Identities of peaks 3-10 were assigned using the NIST database and shown in **(C). (C)** Relative abundance of AaBPS *in-vitro* products for the most active truncated version of the protein (BPS-tr1). **(D)** Relative abundance of selected volatiles in three types of *A annua* Artemis leaves measured by GC-MS: L1-5 (juvenile), L7-9 (expanding), L11-13 (mature). Error bars – SE (n=6).

We cloned the full length and two 5’ truncated forms of the *AaBPS* coding sequence into a plasmid vector for heterologous expression in *E. coli*. Truncation of the putative plastid targeting (PT) sequence was carried out to overcome any possible interference with the production of the protein in the microbial system (**Figure 6A**). Analysis of the *AaBPS* gene using software such as ChloroP or TargetP predicted the 5’ plastid targeting (PT) sequence cleavage site after residue A34 in its amino acid sequence resulting in the truncated AaBPS_tr1 sequence beginning at residue C35. A longer N-terminal PT region in Sage BPS, resulting in the coding region just before the active site lid residues has also been reported (Whittington et al., 2002) and on this basis AaBPS_tr2 was designed to begin at R58, the start of the predicted active site lid residues of *A. annua* BPS. Transformation of *E. coli* BL21 (DE3) resulted in very low levels of soluble full-length protein compared to both truncated versions. All three versions of purified AaBPS protein were subjected to the sage Bornyl diPhosphate Synthase (SoBPS) activity assay using GPP as a substrate with borneol being detected by GC-MS following rAPid alkaline phosphatase treatment of the diphosphate product (Wise et al., 1998). The full‐length and truncated forms of the AaBPS protein all produce borneol [1] as the major product (**Figure 6B**). Unreacted GPP substrates can also be seen hydrolyzed to geraniol [2] (**Figure 6B**). The BPS_tr1 protein is the most active form, producing the most soluble protein as well as turning over 95% of the GPP substrate into borneol and other minor monoterpenes (**Figure 6B**). We have observed a number of minor peaks in the AaBPS-tr1 profile which were absent in the control reaction without AaBPS-tr1 protein added (**Figure 6B**). We used the NIST database to assign these products as described in the Materials and Methods section. Our analysis shows that AaBPS-tr1 is producing 5.6% camphene [9], 0.2% camphene hydrate [4], 2.5% limonene [8], 2.2% α-pinene [10], 1.8% *trans*-sabinene hydrate [5], 0.2% *cis*-sabinene hydrate [7], 0.3% α-terpineol [3] and 0.3% terpinolene [6], in addition to borneol as the major product (86.8%) from GPP substrate (**Figure 6C**).

## Discussion

Our previous work to increase content of the antimalarial drug artemisinin in the medicinal plant *Artemisia annua* L. resulted in creation of F1 and F2 mapping populations and M2 mutagenised populations (Graham et al., 2010). Camphor is a monoterpene, described as one of the major essential oil constituents across *A. annua* varieties. Here we report the discovery of the camphor-null phenotype, apparent in F2 and M2 populations derived from the Artemis F1 variety. The fact the camphor-0 phenotype is not present in F1 plants but appears in F2 populations (**Figure 1**, **S1**), together with results of test crosses of camphor-0 material derived from the M2 population indicating the phenotype is due to a single recessive allele, strongly suggests that the Camphor-0 phenotype is a consequence of segregation of a recessive trait that emerges in the F2 and M2 generations having been acquired from one or other of the parental lines which we previously showed contain a high level of heterozygosity (Graham et al., 2010).

Our results show that camphor accumulates to high concentrations of up to 10% leaf dry weight in Artemis F1 material and that the absence of camphor does not result in an increased flux into artemisinin but rather an increase in artemisinin pathway precursors (**Figure 2A**; **Table S1**). Differential extraction techniques have shown that artemisinin accumulates in sub-apical cavities of GSTs (Duke et al., 1994) and accumulation of other sesquiterpene lactones in sub-apical cavities of GSTs have also been reported across *Artemisia* species (Cappelletti et al., 1986). Although the exact condition in *A. annua* trichomes required for the conversion of DHAA into artemisinin is unknown previous reports strongly suggest such auto-catalytic conversion requires both light and a non-aqueous environment whereas spontaneous transformation of DHAA to DHEDB and deoxyartemisinin appears to be facilitated by a more aqueous environment (Brown and Sy, 2004; Czechowski et al., 2016). One possible explanation of the wild type levels of artemisinin observed in Camphor-0 mutants despite strong increases in FPP and artemisinin precursors is actually that camphor may be an important contributor to the non-aqueous environment required for the conversion of DHAA to artemisinin (**Figure 2D**). In the absence of camphor, we show that in addition to the accumulation of the enzymatically produced artemisinin precursors amorpha-4,11-diene and DHAA, there is also an increase in levels of DHEDB and deoxyartemisinin, possibly as a result of the more aqueous camphor-0 environment favoring non-enzymatic flux of DHAA into these compounds rather than artemisinin (**Figure 2A**; **Table S1**). That artemisinin does still accumulate to wild type levels in the Camphor-0 mutant suggests that to a certain extent the non-aqueous environment generated by other compounds such as non-polar lipids is sufficient for the spontaneous conversion of DHAA to artemisinin. Comparative metabolite profiling of sub-apical cavities versus GST cells, using techniques such as single cell sampling followed by electrospray ionization-mass spectrometry (Nakashima et al., 2016) would shed further light on the *in vivo* chemical environment required for non-enzymatic DHAA conversions and the possible role of camphor in these processes in *Artemisia annua* L.

Detailed metabolite profiling revealed that a number of other lower abundant monoterpenes (relative to camphor) were also missing or strongly reduced in camphor-0 material when compared to camphor - containing Artemis F1, amounting to a reduction in total volatile content of camphor-0 young leaves by 50% (**Figure 2B** and **Table S2**). Levels of sesquiterpenes unrelated to artemisinin were on the other hand elevated in camphor-0 leaf material, which was accompanied by a significant increase in the content of FPP precursor for sesquiterpene synthesis (**Figures 2A, C** and **Table S1**). RNAi-mediated silencing of *amorpha-4,11-diene synthase* (*AMS*) genes resulted in a similar increase in FPP accumulation in transgenic *A. annua* (Catania et al., 2018). That study showed no impact of an increase in FPP on di- or triterpene levels in the silenced lines.

Bioinformatic analysis of the previously published transcriptomics datasets from *Artemisia annua* (Graham et al., 2010) identified *Bornyl diPhosphate Synthase* (*AaBPS*) as a candidate involved in catalyzing the first committed step in camphor biosynthesis in the Artemis F1 hybrid. *AaBPS* is expressed highly in GSTs and its expression was not detectable in camphor-0 individuals (**Figure 3B**). PCR analysis of genomic DNA showed that the *AaBPS* sequence is absent from the genomes of all camphor-0 individuals derived from both F2 and M2 populations but is present in Artemis F1 and the C4 and C1 Artemis parents. -No other sequences present in the transcriptomics datasets are very closely related to *AaBPS*. However, *AaBPS-likeA* and *AaBPS-likeB* were present in genomic DNA from all camphor-0 and camphor-containing material (**Figure S5B**) and *AaBPS-likeA* is also expressed in both camphor-0 and camphor-containing individuals (**Figure 3B** **).** Protein modelling predicts that of the three homologues only AaBPS is likely to be functional on GPP substrate, which is essential for it to act in the first committed step in camphor biosynthesis.

We cloned and functionally characterized the *AaBPS* promoter using promoter-GUS fusions expressed in *Artemisia annua* Artemis F1 and obtained expression patterns consistent with the EST and qRT-PCR analysis of *AaBPS* gene expression (**Figures 3B**; Graham et al., 2010). It is interesting that the *AaBPS* promoter shows similar activity in hair-like (T-shape) non-secretory trichomes on mature leaves and stems (**Figures 4C-E**) but not on the young leaf where its activity seems to be specific to glandular secretory trichomes (**Figures 4A, B**). These results demonstrate the importance of both temporal and spatial regulation of gene expression underpinning production of this most abundant of monoterpenes in *A. annua.*

Heterologous expression of the *AaBPS* sequence in *E. coli* followed by protein purification and *in vitro* bioactivity assays confirm the identity of AaBPS as a BORNYL DIPHOSPHATE SYNTHASE that converts GPP substrate to borneol (**Figure 6B**). The most active version of the protein sequence with plastid targeting (PT) sequence removed (AaBPS-tr1) produced not only borneol (86.8% of the total activity) but also small amounts of other monoterpenes including camphene (5.6%), camphene hydrate (0.2%), limonene (2.5%), α-pinene (2.2%), *trans*-sabinene hydrate (1.8%), *cis*-sabinene hydrate (0.2%), α-terpineol (0.3%) and terpinolene (0.3%) (**Figure 6C**). AaBPS *in vitro* activity on GPP is therefore similar to sage BPS, as SoBPS has been shown to produce borneol (75%), (+)-α-pinene (3.4%), (-)-camphene (9.5%), (+)-camphene (0.5%), (-)-limonene (3.9%), (+)-limonene (3.9%), terpinolene (2.1%), and myrcene (1.5%) (Wise et al., 1998). It is notable that the relative abundance of the minor AaBPS *in vitro* activity products correlate with the abundance of these compounds in all three types of *Artemis* leaf tissues analyzed by GC-MS (**Figure 6D**). It is also noteworthy that some of the minor monoterpenes produced by AaBPS *in vitro* are absent (camphene, *cis-* and *trans-sabinene* hydrate) or strongly reduced (α-pinene) in leaf tissues of camphor-0 line (**Figure 2** and **Table S2**). These results lead us to conclude that AaBPS is not only responsible for production of camphor but also other monoterpenes, amounting to over 50% of total volatiles in *A. annua* F1 hybrid *Artemis* leaves (**Figure 6D** **)**.

## Data availability statement

The datasets presented in this study can be found in online repositories. The names of the repository/repositories and accession number(s) can be found in the article/**Supplementary Material**.

## Authors contributions

TC, CB, AR, DR, DH, TMC, DZ, MS conducted the experiments; TC, CB, DB, IG and PO’m designed experiments, TC, CB, TL, YL, MS, PO’m analyzed the data, TC and IG wrote the article with input from YL, TL and PO’m. All authors contributed to the article and approved the submitted version.

## Funding

This work was supported by The Bill and Melinda Gates Foundation, award number: OPPGH5210. DZ was supported by the China Scholarship Council (No.201909110003).

## Acknowledgments

We thank A. Fenwick, J. Daff, P. Scott, L. Doucet, H. Martin, N. Nattriss, M. Segura, and A. Czechowska for horticulture assistance; G. Chigeza for horticulture management; S. Graham, S. Heywood, B. Kowalik, S. Pandey, R. Simister, and C. Whitehead for laboratory assistance. We thank X. Simonnet and Médiplant for access to the Artemis pedigree.

## Conflict of interest

The authors declare that the research was conducted in the absence of any commercial or financial relationships that could be construed as a potential conflict of interest.

## Publisher’s note

All claims expressed in this article are solely those of the authors and do not necessarily represent those of their affiliated organizations, or those of the publisher, the editors and the reviewers. Any product that may be evaluated in this article, or claim that may be made by its manufacturer, is not guaranteed or endorsed by the publisher.
