# Supplementary material for: Artemisia annua L. plants lacking Bornyl diPhosphate Synthase reallocate carbon from monoterpenes to sesquiterpenes except artemisinin
Source: Front Plant Sci. 2022 Oct 12;13:1000819. doi: 10.3389/fpls.2022.1000819 (PMC9597464; doi:10.3389/fpls.2022.1000819)
Supplement: Supplementary file 1 [file DataSheet_1.docx]

Supplementary Material

Flux attenuation in *Artemisia annua* L. plants lacking *Bornyl diPhosphate Synthase* suggests a role for monoterpenes in controlling artemisinin production

**Tomasz Czechowski^1^, Caroline Branigan^1^, Anne Rae^1^, Deborah Rathbone^1^, Tony R. Larson^1^, David Harvey^1^, Theresa Catania^1^, Dong Zhang^1^, Yi Li^1^, Melissa Salmon^2^, Dianna J. Bowles^1^, Paul O´Maille^2^ and Ian A. Graham^1,*^**

^1^ Centre for Novel Agricultural Products, Department of Biology, University of York, Heslington, York YO10 5DD, United Kingdom; ^2^ Department of Metabolic Biology, John Innes Centre, Norwich Research Park, Norwich NR4 7UH, United Kingdom.

*** Correspondence:**Ian A. Graham
[ian.graham@york.ac.uk](mailto:ian.graham@york.ac.uk)

# Supplementary Figures and Tables

## Supplementary Tables

Table S1. Levels of 37 UPLC-MS detected metabolites for three different leaf types extracted from Artemis F1 and Camphor-0 M3.

Table S2. Levels of 36 GC-MS detected metabolites for three different leaf types extracted from Artemis F1 and Camphor-0 M3.

Table S3. Primer sets used in this study.

## Supplementary Figures


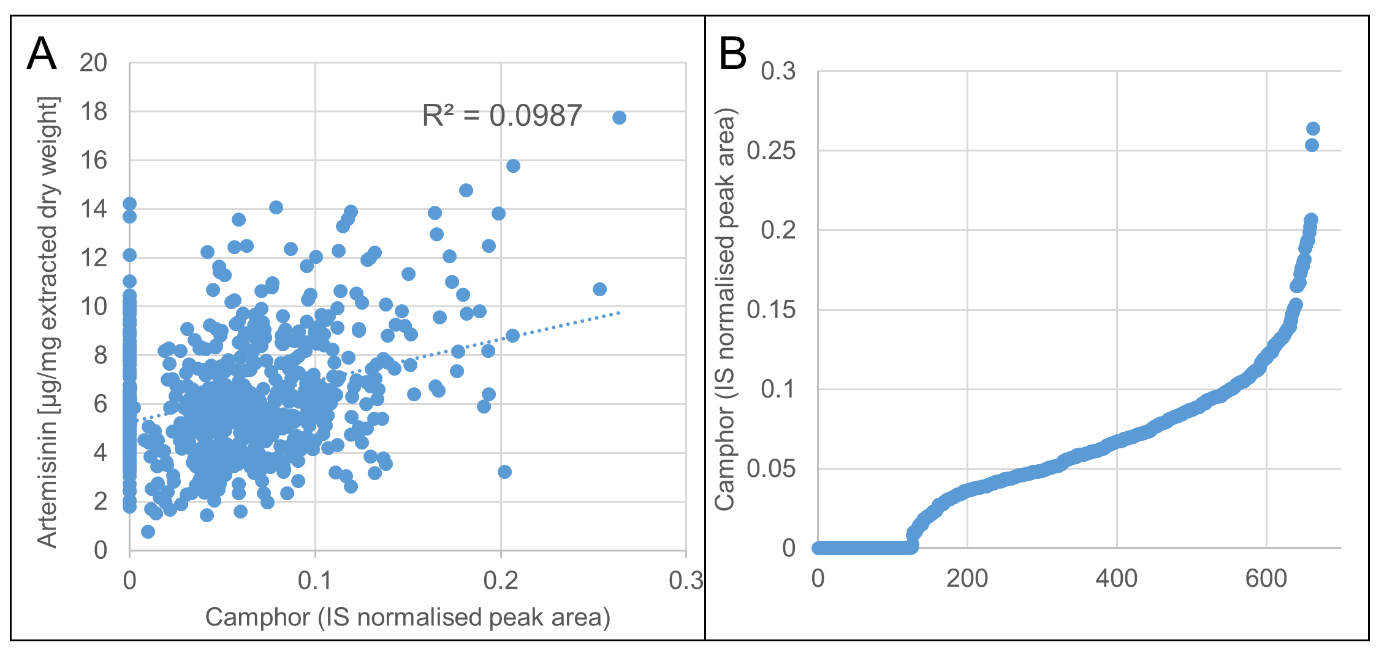


**Fig. S1 Artemisinin and camphor concentration in F2 mapping population**

Content of camphor and artemisinin analysed in extracts from fresh L11-13 mature leaves of Artemis F2 population grown for 12 weeks in glasshouse. LC-MS analysis performed on 662 progeny of 85 randomly chosen self-pollinated Artemis F1 individuals using 3 technical replicates


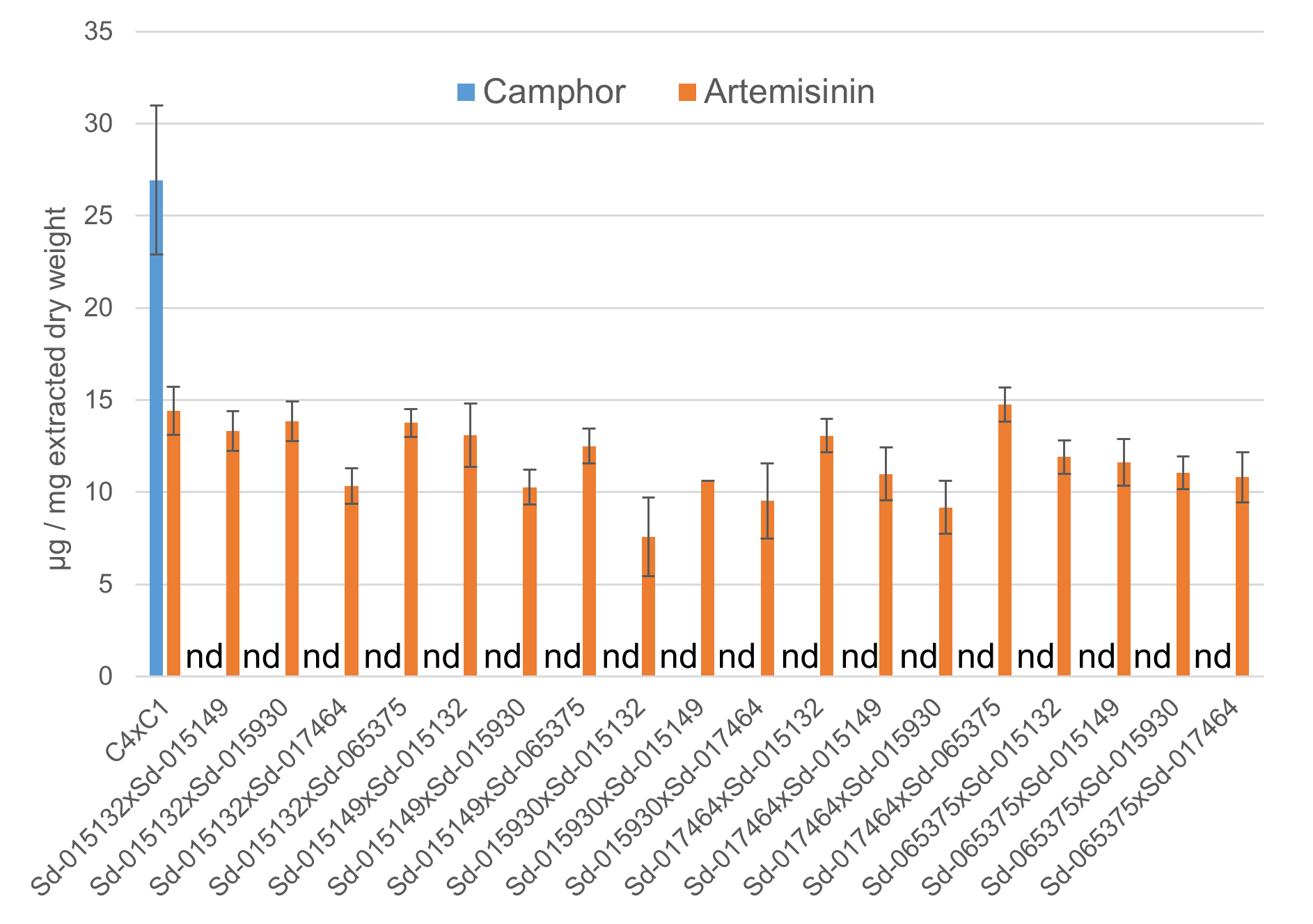


**Fig. S2 Camphor and artemisinin concentrations in Artemis F1 and progeny of camphor-0 M2 test crosses.**

Camphor and artemisinin content was analysed for fresh L11-13 mature leaves of glasshouse grown progeny of Artemis F1 (C4 x C1 cross) and M3 progeny of crosses between six selected M2 camphor-0 individuals.  Camphor and artemisinin content was quantified using genuine standards by GC- and LC-MS respectively, as described in the Materials and Methods. nd - not detectable Error bars SE (n=5-10)


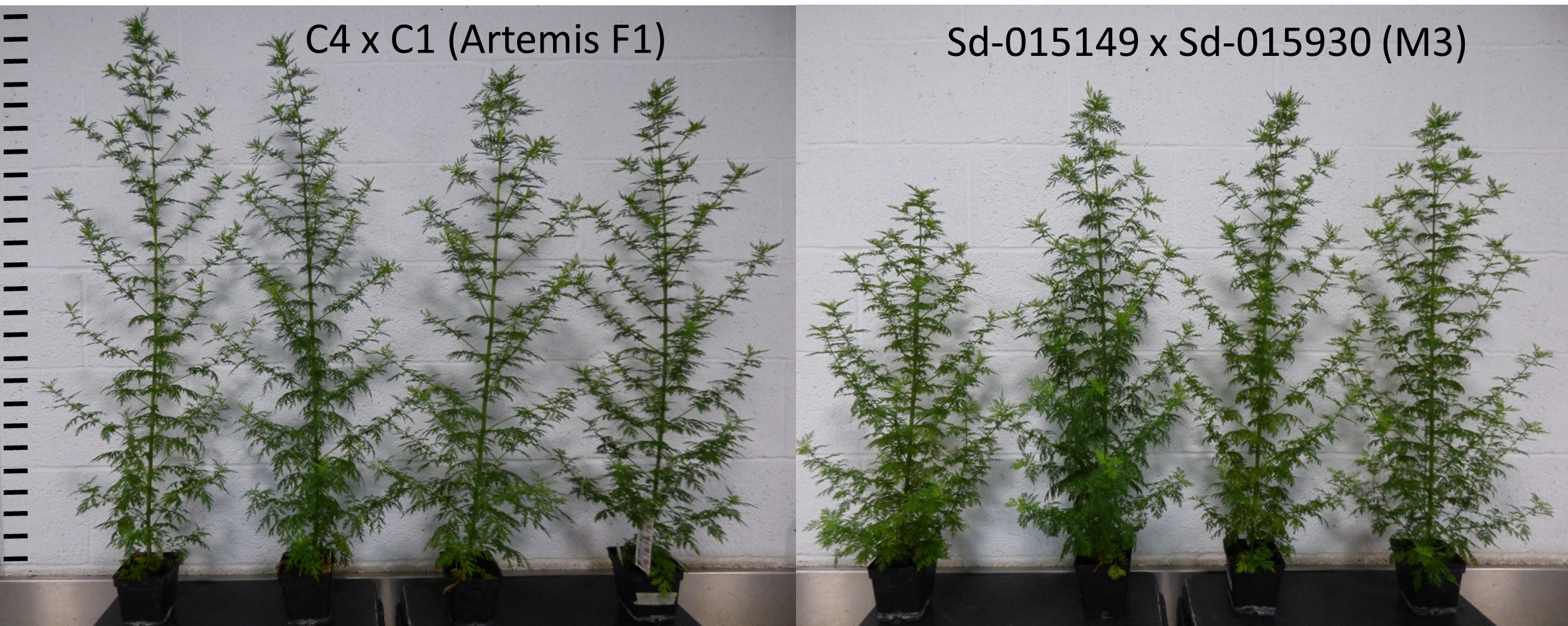


**Fig. S3 Morphology of Artemis F1 and progeny of camphor-0 M2 test cross.** Morphology of glasshouse-grown 12-week old Artemis F1 and progeny of a one M2 test cross, selected from the set presented on Fig. S2. Scale bar on the left - 5cm.


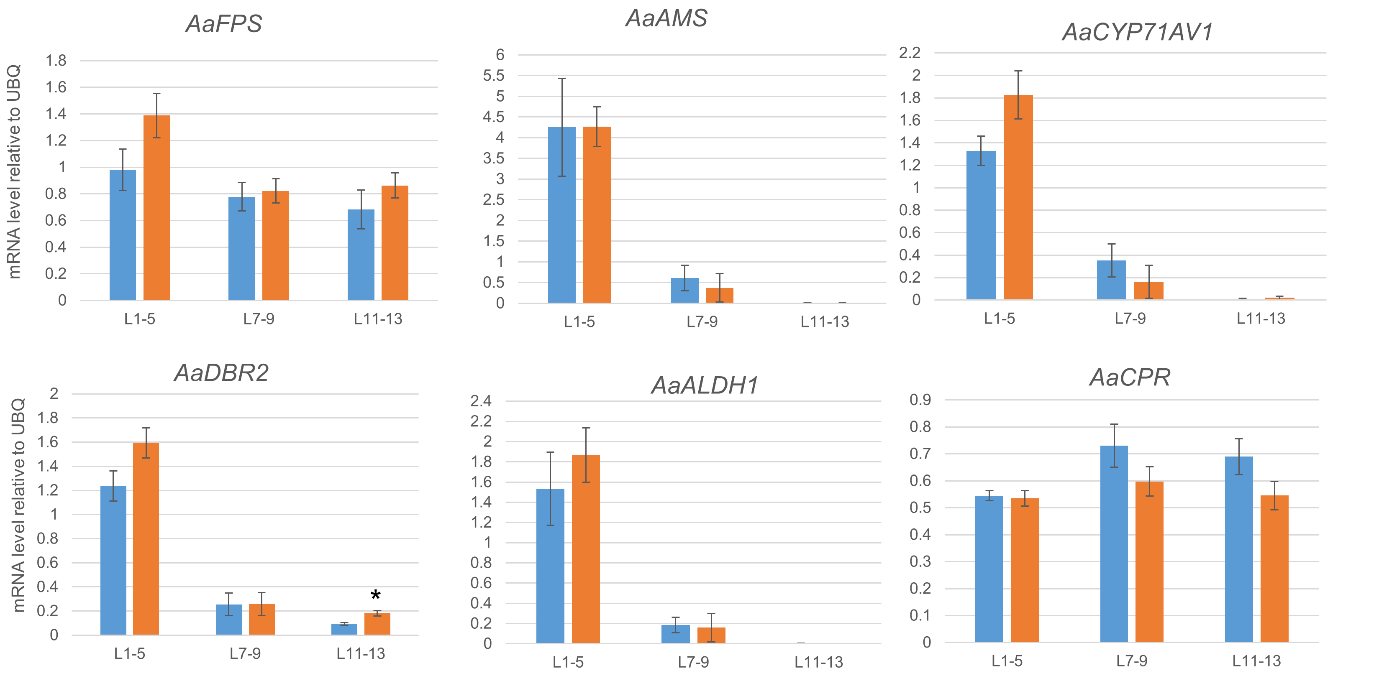


**Fig. S4 qRT-PCR quantified expression level of artemisinin-pathway genes in camphor-0 lines.**

qRT-PCR profiling of transcripts encoding enzymes of the artemisinin biosynthetic pathway.  mRNA was extracted from L1-5 (juvenile), L7-9 (expanding) and L11-13 (mature) leaves as counted from the apical meristem from 12-weeks old glasshouse-grown Artemis F1 (blue bars) and selected camphor-0 M3 (orange bars) as described in materials and methods. Error bars – SE (n=9). Asterisk indicates statistically significant difference (t-test determined) between Artemis and camphor-0 at p<0.05. Gene abbreviations: *AaFPS*- Farnesyl diPhosphate Synthase, *AaAMS* – amorpha-4,11-diene synthase, *AaCYP71AV1* - amorpha-4,11-diene C-12 oxidase, *AaDBR2* - artemisinic aldehyde Δ 11 (13) reductase, *AaALDH1* - aldehyde dehydrogenase,  *AaCPR* – cytochrome P450 reductase.


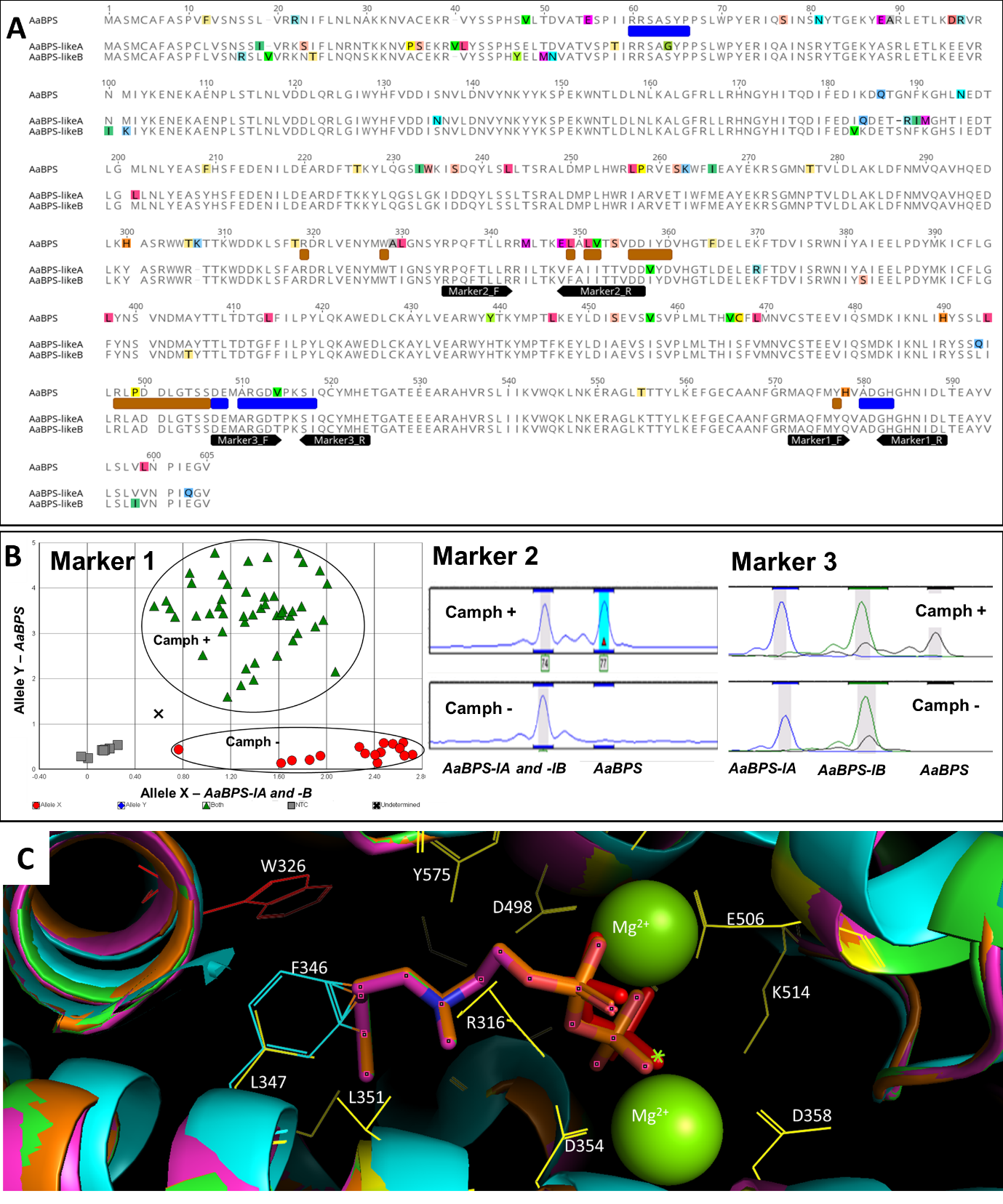


**Fig. S5 Identification of *AaBPS* homologous sequences in camphor-0 and camphor-containing individuals.**

**(A)** Alignment of cDNA – predicted  amino acid sequences of AaBPS and its two closest homologues:  AaBPS-likeA and AaBPS-likeB. Blue bar – putative active site lid residues, Brown bar – substrate binding site residues, Black bars  – position of PCR primer pairs for three molecular markers used for genotyping. **(B)** Example of genotyping results for Artemis F2 camphor-0 (Camph-) and camphor-containing (Camph +) individuals using three DNA markers shown in panel A. Genomic DNA was extracted from Artemis F2 population individuals segregating in camphor-containing (camph +) and  camphor-0 (camph -) as described in materials and methods. KASPar genotyping assays were performed for Marker 1 as described in materials and methods and results for 65 Artemis F2 individuals. SNP genotyping assays were performed on the ABI3730xl platform as described in materials and methods and the example results for two selected Artemis F2 individuals  are shown for Marker 2 and Marker 3. **(C)** Overlay of the putative catalytic and substrate binding site of the cDNA-deduced amino acid sequences for AaBPS, AaBPS-likeA and AaBPS-likeB with SoBPS structure (1N1B). cDNA-deduced amino acid sequences for AaBPS (green), AaBPS-likeA (orange) and AaBPS-likeB (magenta) were used for protein modelling using the I-TASSER approach. Models with the lowest C-score were overlaid with SoBPS (1n22, cyan) structure with Mg2+ (green spheres) and GPP (coloured licorice sticks) bound  using PyMOL software. I-TASSER predicted substrate (GPP) binding residues (yellow) and catalytic W326 (red) are shown for AaBPS. F346 present in AaBPS-likeA and AaBPS-likeB is highlighted  in cyan.
